# A pilot study of improvised CPAP (iCPAP) via face mask for the treatment of adult respiratory distress in low-resource settings

**DOI:** 10.1186/s12245-019-0224-0

**Published:** 2019-03-05

**Authors:** Brendan H. Milliner, Suzanne Bentley, James DuCanto

**Affiliations:** 1 Division of Emergency Medicine, University of Utah, 30 N 1900 E 1C026, Salt Lake City, UT 84132 USA; 2 Simulation Center at Elmhurst and Department of Emergency Medicine, Elmhurst Hospital Center, Elmhurst, NY USA; 3 Departments of Emergency Medicine and Medical Education, Icahn School of Medicine at Mount Sinai, 3 East 101st Street, Box 1620, New York, NY 10029 USA; 4 Department of Anesthesiology, Aurora St. Luke’s Medical Center, 2900 W Oklahoma Ave, Milwaukee, WI 53215 USA

**Keywords:** Continuous positive airway pressure, Emergency airway management, Critical care, Low-resource settings

## Abstract

**Background:**

Continuous positive airway pressure (CPAP) is a mode of non-invasive ventilation used to treat a variety of respiratory conditions in the emergency department and intensive care unit. In low-resource settings where ventilators are not available, the ability to improvise a CPAP system from locally available equipment would provide a previously unavailable means of respiratory support for patients in respiratory distress. This manuscript details the design of such a system and its performance in healthy volunteers.

**Methods:**

An improvised CPAP system was assembled from standard emergency department equipment and tested in 10 healthy volunteers (6 male, 4 female; ages 29–33). The system utilizes a water seal and high-flow air to create airway pressure; it was set to provide a pressure of 5 cmH2O for the purposes of this pilot study. Subjects used the system in a monitored setting for 30 min. Airway pressure, heart rate, oxygen saturation, and end-tidal CO2 were monitored. Comfort with the device was assessed via questionnaire.

**Results:**

The system maintained positive airway pressure for the full trial period in all subjects, with a mean expiratory pressure (EP) of 5.1 cmH2O (SD 0.7) and mean inspiratory pressure (IP) of 3.2 cmH2O (SD 0.8). There was a small decrease in average EP (5.28 vs 4.88 cmH2O, *p* = 0.03) and a trend toward decreasing IP (3.26 vs 3.07 cmH2O, *p* = 0.22) during the trial. No significant change in heart rate, O2 saturation, respiratory rate, or end-tidal CO2 was observed. The system was well tolerated, ranked an average of 4.0 on a 1–5 scale for comfort (with 5 = very comfortable).

**Conclusions:**

This improvised CPAP system maintained positive airway pressure for 30 min in healthy volunteers. Use did not cause tachycardia, hypoxia, or hypoventilation and was well tolerated. This system may be a useful adjunctive treatment for respiratory distress in low-resource settings. Further research should test this system in settings where other positive pressure modalities are not available.

**Electronic supplementary material:**

The online version of this article (10.1186/s12245-019-0224-0) contains supplementary material, which is available to authorized users.

## Background

Continuous positive airway pressure (CPAP) is commonly used as a means of respiratory support in patients with respiratory distress in both hospital and pre-hospital settings [1–3]. CPAP has been shown to reduce symptom burden, decrease intubation rates, and may reduce mortality rates [4–6]. In US hospitals, CPAP involves the use of a mechanical ventilator or purpose-built CPAP device to generate airway pressure and regulate the respiratory cycle. However, in many low- and middle-income countries (LMIC) and global health settings, ventilators and CPAP devices may not be readily available. Respiratory distress and respiratory failure have been identified as key areas in which the implementation of technology in low-resource settings has not been sufficient to meet the needs of patients [7, 8], and an alternative means of providing CPAP would help to address this unmet need.

The pediatric literature describes “Bubble CPAP,” a low-tech means of generating airway pressure by bubbling expired air or oxygen through a fixed amount of water. Bubble CPAP is delivered via nasal prongs and has been successfully used in neonatal respiratory distress both in US hospitals and in low-resource settings [9–12]. This approach has not been studied for use in adult respiratory distress.

The respiratory setup described in this manuscript (termed “improvised CPAP” or iCPAP) is based on the principle of bubble CPAP and intended as a means of providing adult CPAP when a ventilator is not available. The system, detailed below, combines a respiratory mask and tubing with a high-flow source of medical air or oxygen and a water seal to generate airway pressure. Due to its simplicity, the system can be assembled out of basic respiratory equipment. This manuscript describes a pilot study of the iCPAP system in healthy volunteers to determine its ability to reliably generate positive pressure and to provide initial safety data.

## Methods

### Subjects

Ten healthy emergency medicine residents were included. Six participants (60%) were male; the average age was 30 years (range 29–33). Volunteers with active pulmonary disease, a history of pneumothorax, severe claustrophobia, and pregnant subjects were excluded.

### Device design and operation

**Fig. 1:**
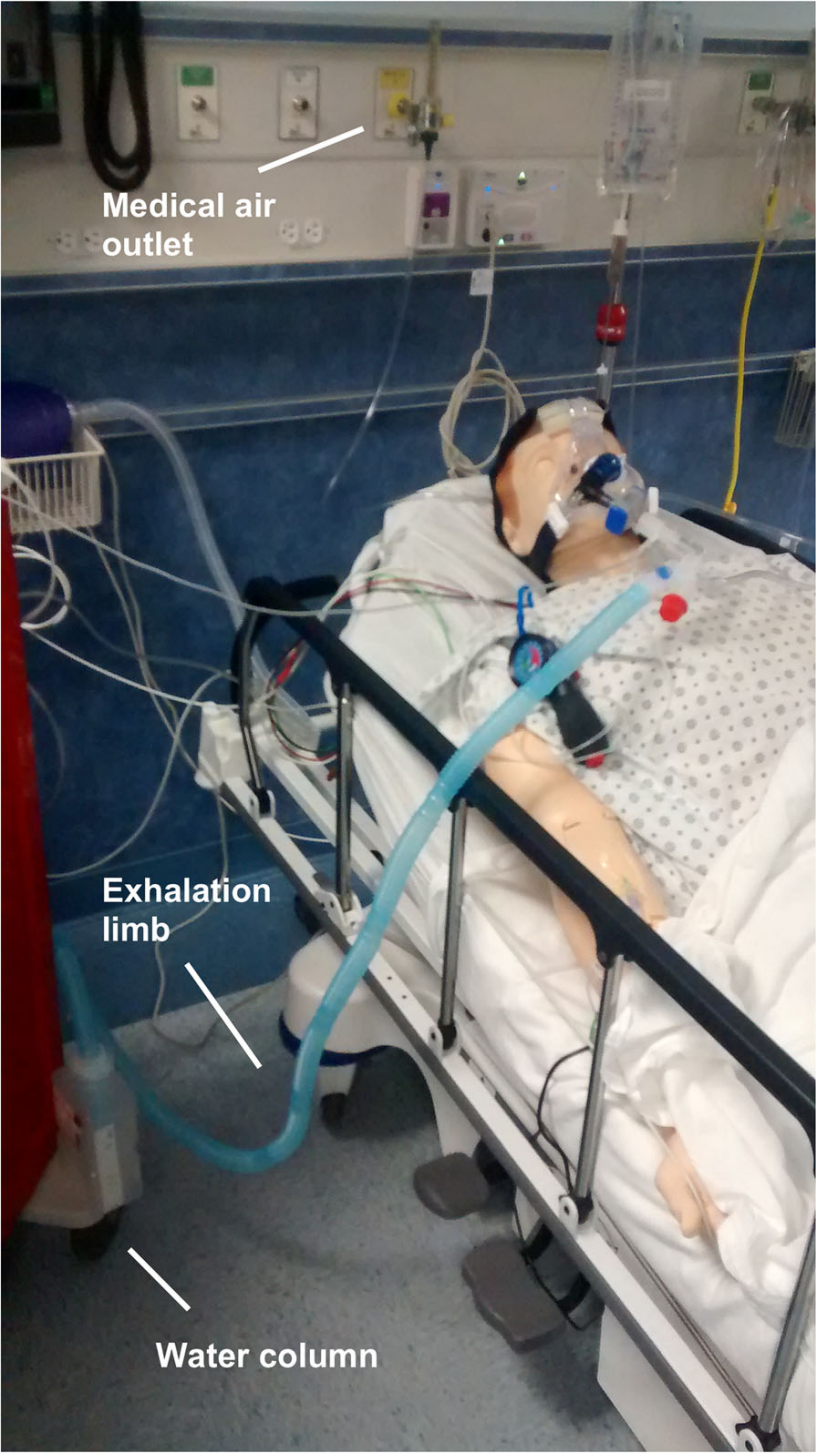
Overview of the device. The iCPAP device has been fitted to a simulation mannequin with basic components labeled

**Fig. 2:**
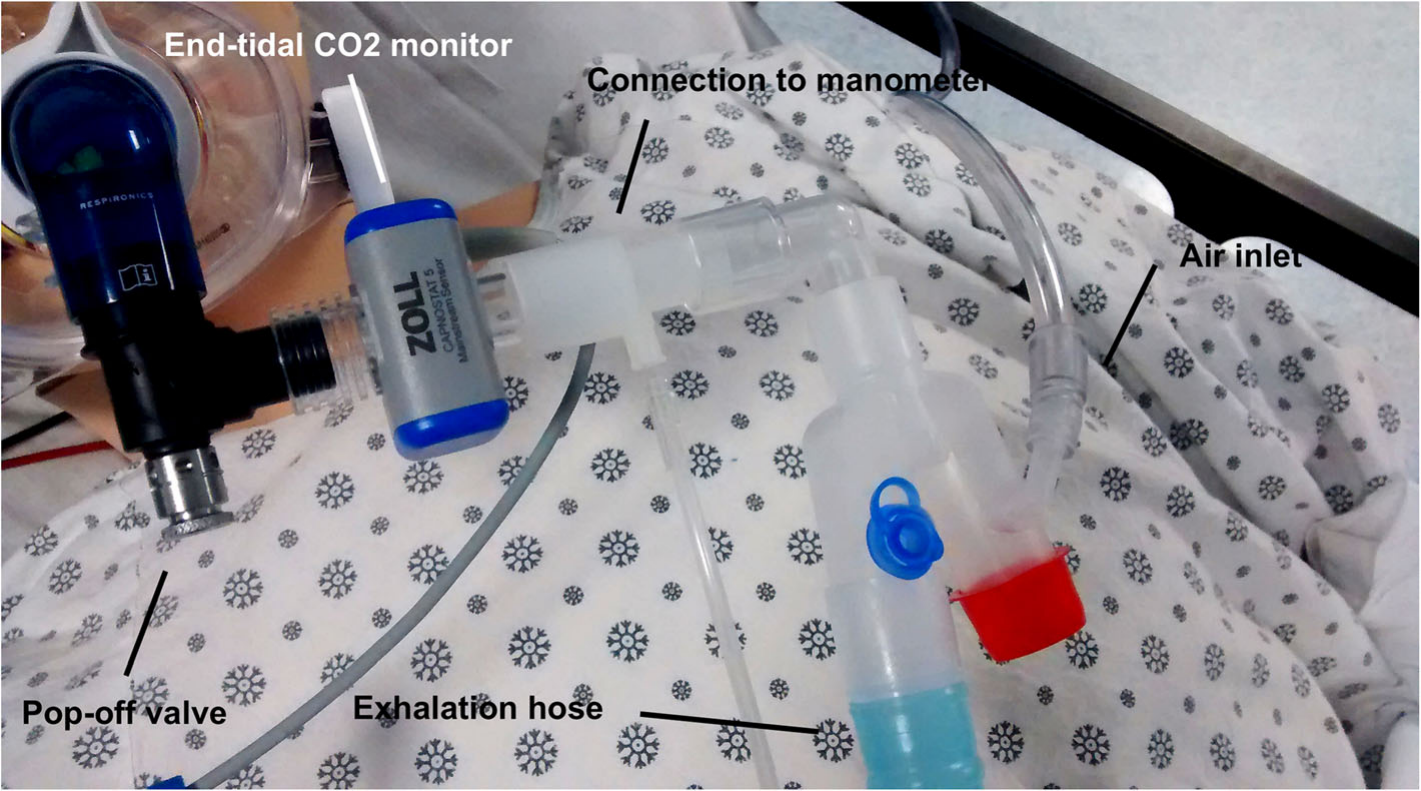
Enhanced view of the mask and attached components

A respiratory face mask was attached to a Y-shaped airway connector; one end of the connector was capped while the other was connected to a 6-ft length of airway tubing submerged in a container of sterile water. High-flow medical air at 30 l/min was piped into the system near the capped connector using a separate port. For the purposes of this trial, a respiratory pop-off valve, an end-tidal CO_2_ monitor, and a laptop-based airway pressure measurement device (Phidgets, Inc.) were interposed between the face mask and the water reservoir (see Figs. 1 and 2).

**Fig. 3:**
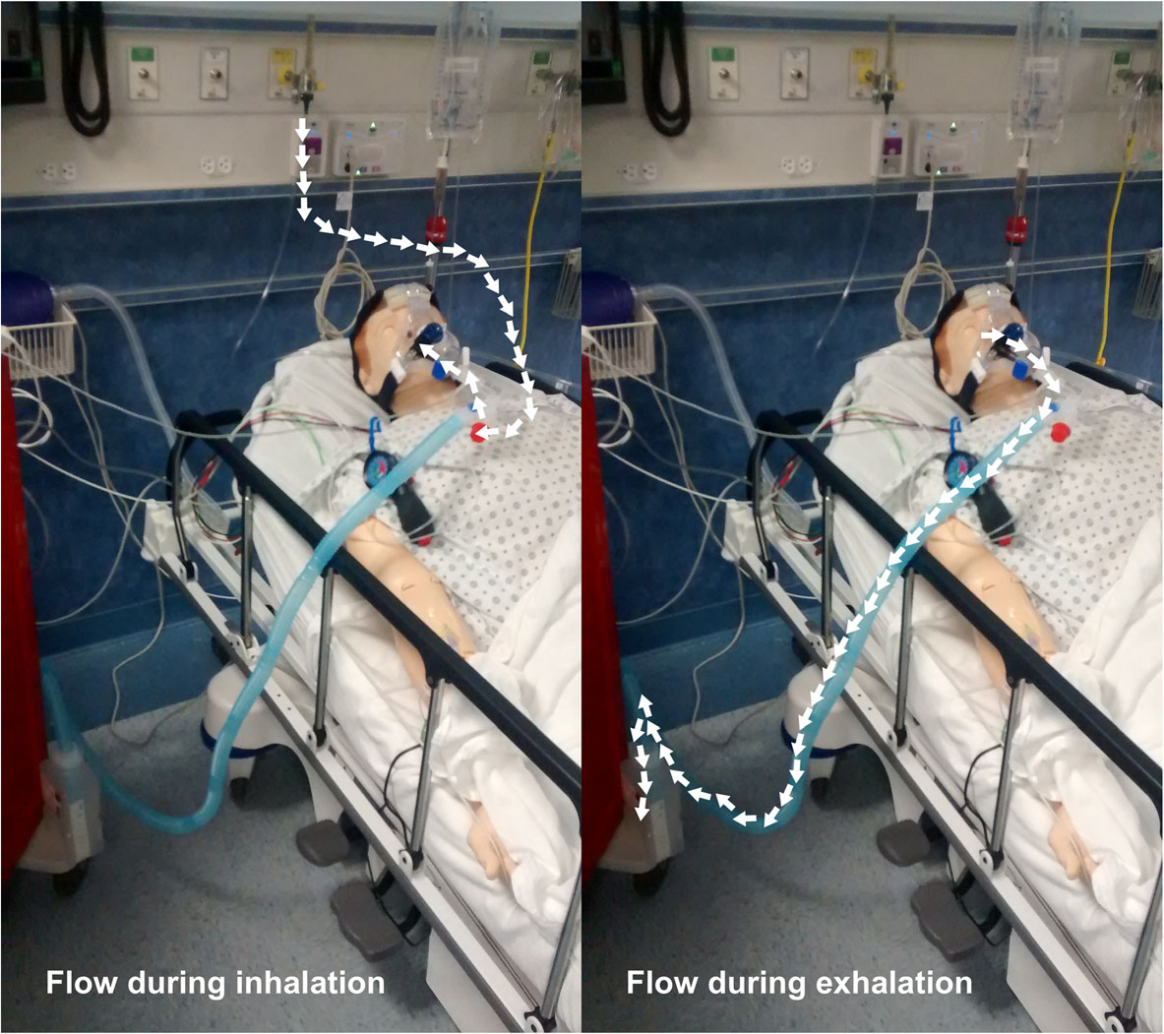
Flow patterns. During inhalation, air flows from the wall outlet to the mask. During exhalation, air travels from the patient through the exhalation limb to the reservoir

During inhalation, positive pressure in the iCPAP system is created by the rapid inflow of air from the wall outlet. During exhalation, the force required to exhale through water in the reservoir creates positive expiratory pressure (Fig. 3). For the purposes of the current study, the exhalation tubing was fixed 5 cm below the surface of the reservoir, generating 5 cmH2O of expiratory pressure; depth can be adjusted to vary the supplied pressure.

### Device trials

Device trials were carried out in a monitored simulation center environment. Subjects wore the iCPAP setup during normal breathing for 30 min. Airway pressure data were continuously recorded at 0.1 s intervals. Subjects’ heart rate (HR) and oxygen saturation (SpO_2_) were continuously monitored and recorded every minute, while end-tidal CO_2_ (EtCO_2_) was recorded at 5-min intervals. At the conclusion of the trial, each subject completed a brief questionnaire evaluating his or her experience with the device (Additional file 1).

### Data analysis

The visual programming software Max (Cycling ‘74) was used for pressure data sampling. Raw pressure data was separated into inhalation and exhalation phases based on the average slope of several adjacent data points; this was further refined visually using a graph of the pressure waveform. Mean inspiratory pressure (IP), expiratory pressure (EP), and respiratory rate (RR) during the first 5 min and the last 5 min of each trial were calculated in Microsoft Excel. Paired *t* tests were used for analysis of significance.

Trends in subjects’ HR, SpO_2_, and EtCO_2_ throughout the trial session were analyzed for significance with repeated-measures ANOVA (IBM SPSS). The post-trial questionnaire was analyzed using numerical averages of each Likert scale item.

## Results

**Fig. 4:**
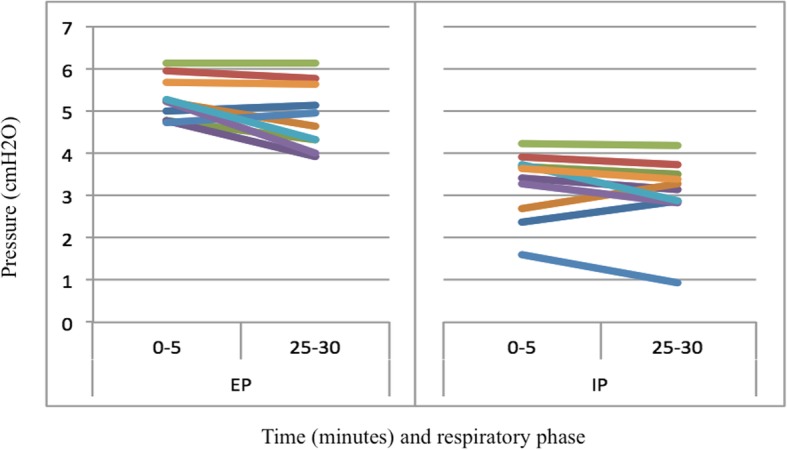
Airway pressure over time. Graphs reflect expiratory pressure (EP) and inspiratory pressure (IP) during the sampled periods within the first 5 min (0–5) and last 5 min (25–30) of each trial. Each line represents a single participant

**Fig. 5:**
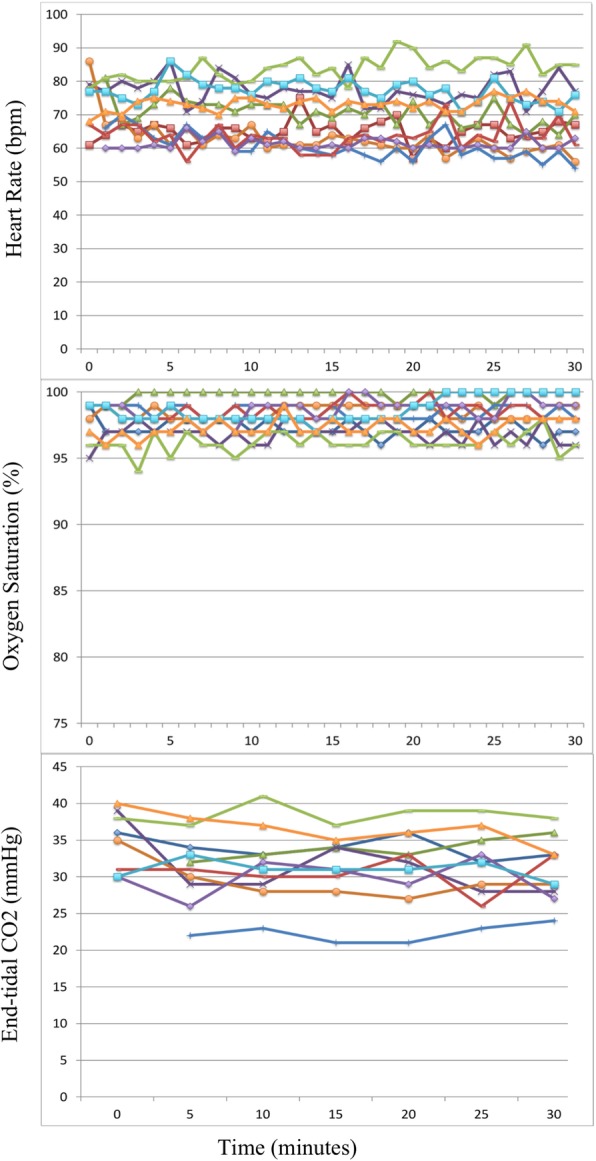
Heart rate, oxygen saturation, and end-tidal CO2 versus time. Each line represents a single participant

The system maintained positive airway pressure for the full trial period in all subjects, with mean EP of 5.1 cmH2O (SD 0.7) and mean IP of 3.2 cmH2O (SD 0.8) over both measured time periods. A small but significant decrease in mean expiratory pressure was observed over the course of the trial (5.3 vs 4.9 cmH2O, *p* = 0.03), as well as a trend toward decreasing inspiratory pressure (3.3 vs 3.1 cmH2O, *p* = 0.22; Fig. 4). No significant effect of time on HR (*F* [29,261] = 0.915, *p* = 0.595), SpO_2_ (*F* [29,261] = 0.976, *p* = 0.505), or EtCO_2_ (*F* [5,45] = 0.208, *p* = 0.958) was seen during the trial (Fig. 5). There was no significant difference in mean RR between the two measured time periods (14.1 vs 15.5 breaths per minute, *p* = 0.2).

The system was ranked an average of 4.0 on a 1–5 scale for overall comfort (with 5 = very comfortable).

## Conclusions

The majority of recent global health efforts have concentrated on population-based care such as maternal and child health and control of communicable disease. While this is of the utmost importance, it does not address the separate concern of providing care to those patients who become critically ill in low-resource settings [7, 8].

In this pilot study of an improvised CPAP system, positive pressure ventilation was sustained in healthy volunteers over a period of 30 min without any detectable CO_2_ retention or significant changes in subjects’ vital signs. The system was well tolerated by all study participants. A small decrease in airway pressure was observed throughout the course of the trial, which may be explained by mask leak or splashing of water out of the pressure reservoir. While the iCPAP system is similar in principle to neonatal bubble CPAP, it poses theoretical challenges as the adult face mask renders it a closed system with the potential risk of CO_2_ retention or dangerous airway overpressure. This study provides an encouraging proof-of-concept for this approach. While the current study examined a relatively low PEEP value of 5 cmH2O, the pressure administered can be increased by fixing the exhalation limb at a greater depth. Limited experimentation with higher pressure values suggests that 8.0 cmH2O of PEEP can be reliably maintained by the device; we did not attempt to increase the pressure above this level.

As a pilot study, this trial does not address the potential challenges of implementing the iCPAP system in a field setting. One challenge is the flow of air required to generate airway pressure, as many LMIC hospital settings may not have a centralized air pressure system. A low-cost neonatal bubble CPAP system has been successfully implemented in Ghana using aquarium air pumps to generate the required pressure [13, 14], and higher-output ambient air pumps could be used in a field-ready iCPAP device, such as oil-free air compressor pumps utilized for powering tools and inflating vehicle tires.

**Fig. 6:**
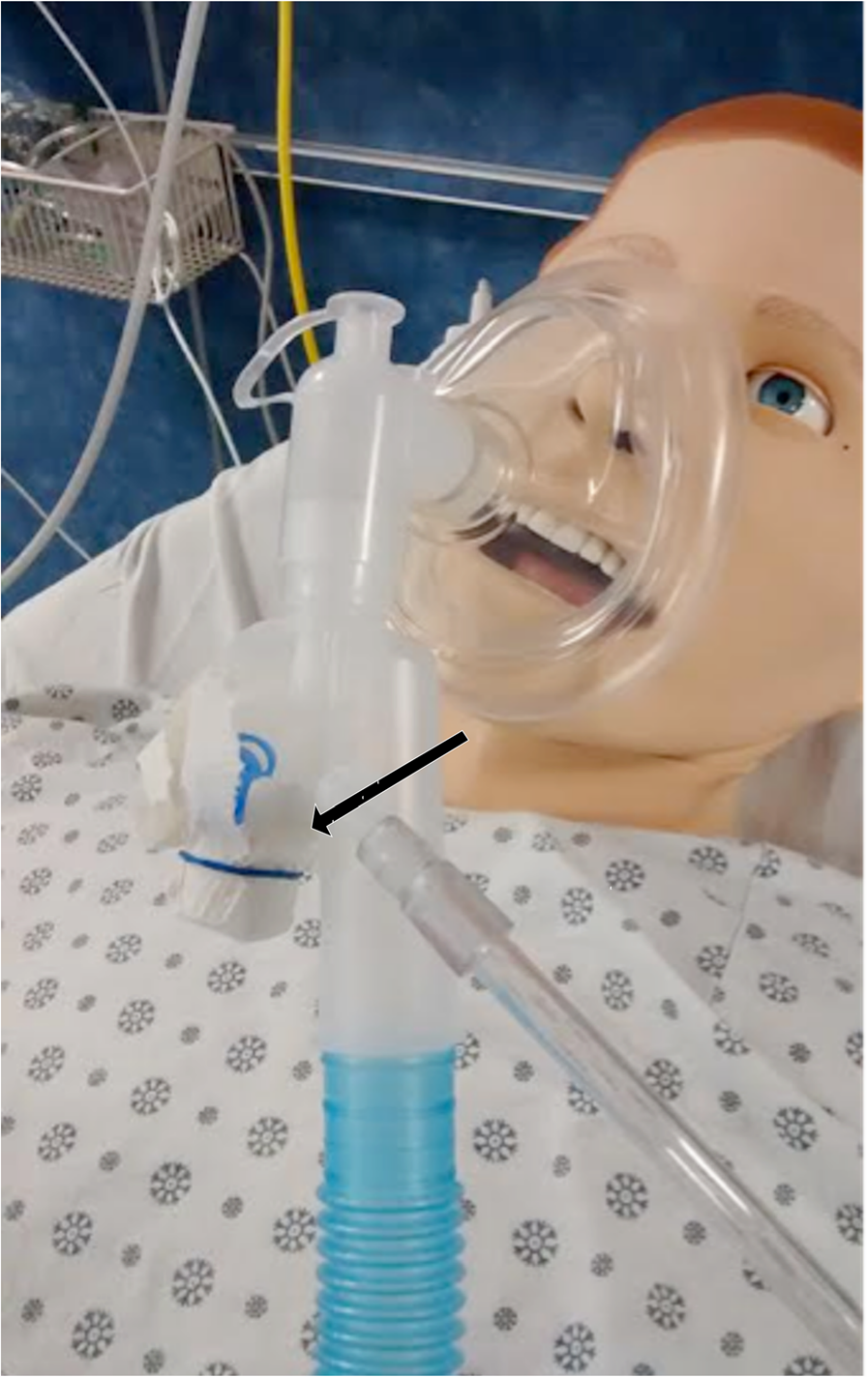
Model of low-tech pressure-limiting system. The cap on the unused Y connector has been replaced with a paper cover (arrow). Monitoring equipment has been omitted for the sake of clarity

Another challenge is the availability of the face mask and pop-off valve used in constructing the system. A simple anesthesia face mask fitted with a head harness can be used if alternative masks are not available; this setup is frequently used for pre-oxygenation prior to surgical procedures. Regarding the pop-off valve, limited experimentation suggests that a simple alternative can be constructed using a piece of tissue paper attached with a rubber band to the unused end of the system’s Y connector (Fig. 6); this or similar alternatives may be further explored depending on locally available materials.

Further studies should trial the iCPAP system in patients with respiratory distress in resource-limited settings where ventilator-based CPAP is not available. Measurement of end-tidal CO_2_ or blood pCO_2_ should be conducted during initial field studies as the risk of CO_2_ retention in a patient with a rapid respiratory rate remains unknown.

## Additional file

Additional file 1: Post-trial questionnaire. (DOCX 13 kb)

## Abbreviations

CPAP: Continuous positive airway pressure
EP: Expiratory pressure
etCO2: End-tidal carbon dioxide
HR: Heart rate
iCPAP: Improvised continuous positive airway pressure
IP: Inspiratory pressure
RR: Respiratory rate
SpO2: Oxygen saturation
