# Supplementary material for: A pilot study of improvised CPAP (iCPAP) via face mask for the treatment of adult respiratory distress in low-resource settings
Source: Int J Emerg Med. 2019 Mar 5;12:7. doi: 10.1186/s12245-019-0224-0 (PMC6399909; doi:10.1186/s12245-019-0224-0)
Supplement: Supplementary file 1 — Post-trial questionnaire. (DOCX 13 kb) [file 12245_2019_224_MOESM1_ESM.docx]

**Additional file 1: Post-trial questionnaire**

**Post-trial Questionnaire**

How comfortable did you feel overall during your use of the CPAP system? (1 – not at all comfortable; 5 – very comfortable)

1 2 3 4 5

To what degree did you experience any of the following feelings? (1 – not at all; 5 – strongly)

Claustrophobia

1 2 3 4 5

Anxiety

1 2 3 4 5

Breathlessness

1 2 3 4 5

Dizziness

1 2 3 4 5

Do you have any prior personal experience using CPAP devices?

Do you have any additional feedback you wish to share with the research team? (write below)
